# Supplementary material for: The ecology and quantitative genetics of seed and seedling traits in upland and lowland ecotypes of a perennial grass
Source: Evol Lett. 2022 Nov 22;6(6):460–73. doi: 10.1002/evl3.297 (PMC9783394; doi:10.1002/evl3.297)
Supplement: Supplementary file 1 — Supplementary file S1 [file EVL3-6-460-s006.docx]

**Supplementary file S1:**

**Phenotypic traits measurement at the seed, germination and earlier growth stage**

**Seed mass**

We weighted 100 seeds per line using an analytical balance (Mettler Toledo, Columbus, Ohio, USA) in three sets. Seed mass was calculated for each line dividing total mass by 100 (Seed weight= weight of 100 seeds / 100).

**First day of germination and germination percentage**

We inspected petri dishes once a day (starting at 11am) to record germination. Germination timing for each genotype was recorded as the first day that germination was observed from a petri dish. We recorded the number of germinated seedlings from every petri dishes at ten days after germination time. We calculated the germination rate as the per genotype following the equations below:

Germination Percentage = ((number of seedlings/ 15 seeds) X 100).

**Shoot length and root length**

We unsealed the petri dishes five days after germination and measured shoot length with a metric scale on three randomly selected seedlings per line. Then, we sprayed the upper lid of the petri dishes and resealed them with parafilm. While measuring the seedlings at five days, we marked these seedlings to identify them for subsequent measurement. Five days later, we again measured shoot and root length from the same marked seedlings (ten days after germination).
