# Supplementary material for: The ecology and quantitative genetics of seed and seedling traits in upland and lowland ecotypes of a perennial grass
Source: Evol Lett. 2022 Nov 22;6(6):460–73. doi: 10.1002/evl3.297 (PMC9783394; doi:10.1002/evl3.297)
Supplement: Supplementary file 7 — Supplementary file S7 [file EVL3-6-460-s003.docx]

**Supplementary file S7**

Genomic positions and effects of the seed biology associated QTL identified in the RIL population. Main and epistatic effects of the identified QTLs have been listed here along with their maximum QTL LOD score, position on linkage map, percent of variance explained by each QTL, total variance explained by each QTL model, and the positive allele donor for each QTL.

| **Trait** | **Chr** | **Pos (cM)** | **LOD Peak** | **1.5-LOD Interval** | **%Var** | **Effect** | **SE** | **%**  **model**  **variation** | **Donor** |
| --- | --- | --- | --- | --- | --- | --- | --- | --- | --- |
| SM | 1 | 97.7 | 3.50 | 48-123 | 3.9 | 0.030 | 0.007 | 31.90 | HAL2 |
| SM | 2 | 85.3 | 6.00 | 82-96 | 6.7 | 0.041 | 0.007 |  | HAL2 |
| SM | 3 | 86.3 | 4.73 | 22-92 | 5.3 | 0.036 | 0.007 |  | HAL2 |
| SM | 5 | 138.6 | 4.50 | 129-142 | 5.0 | 0.038 | 0.008 |  | HAL2 |
| SM | 7 | 71.3 | 5.79 | 61-82 | 6.5 | 0.040 | 0.007 |  | HAL2 |
| GP | 2 | 26.3 | 3.1 | 20-36 | 3.4 | -3.828 | 1.010 | 36.05 | FIL2 |
| GP | 2 | 83.9 | 8.11 | 81-85 | 9.2 | -6.389 | 1.021 |  | FIL2 |
| GP | 3 | 118.9 | 4.72 | 108-125 | 5.21 | -4.647 | 0.987 |  | FIL2 |
| GP | 5 | 136 | 3.80 | 130-138 | 4.1 | -4.827 | 1.148 |  | FIL2 |
| GP | 9 | 128.1 | 6.46 | 121-129 | 7.2 | -6.329 | 1.142 |  | FIL2 |
| SL5D | 1 | 108 | 3.93 | 100-118 | 4.9 | -0.670 | 0.156 | 26.45 | FIL2 |
| SL5D | 1 | 25.7 | 4.75 | 21-28 | 6.0 | -0.715 | 0.151 |  | FIL2 |
| SL5D | 2 | 131.8 | 4.22 | 122-143 | 5.3 | 0.678 | 0.152 |  | HAL2 |
| SL5D | 4 | 47.1 | 4.11 | 42-61 | 5.2 | 1.702 | 0.160 |  | HAL2 |
| SL5D | 7 | 82.9 | 5.23 | 66-82 | 6.6 | 0.772 | 0.155 |  | HAL2 |
| SL10D | 2 | 126 | 6.19 | 120-134 | 9.7 | 0.073 | 0.013 | 9.75 | HAL2 |
| RL10D | 3 | 11.7 | 3.82 | 10-21 | 6.1 | 0.152 | 0.036 | 6.13 | HAL2 |
| GT | 6 | 50 | 7.64 | 44-53 | 11.5 | -0.797 | 0.161 | 15.19 | FIL2 |
| GT | Epi6:9 | NA | 7.46 | NA | 11.1 | -0.983 | 0.163 |  | NA |
| GT | 9 | 121.3 | 9.90 | 116-125 | 15.1 | 0.928 | 0.156 |  | HAL2 |

Chr, linkage group; Pos (cM), Centimorgan position of the QTL peak; LOD, logarithm of odds; 1.5-LOD Interval, confidence interval at 1.5 LOD drop; %Var, percent of variance explained by each QTL; SE, standard error for each identified QTL; Effect, QTL effects; %model var, percent of variance explained by each QTL model for traits; Donor, positive allele donor for identified QTLs; Epi, Epistasis.
